# Assessment and prediction of spatial patterns of human-elephant conflicts in changing land cover scenarios of a human-dominated landscape in North Bengal

**DOI:** 10.1371/journal.pone.0210580

**Published:** 2019-02-01

**Authors:** Dipanjan Naha, S. Sathyakumar, Suraj Dash, Abhishek Chettri, G. S. Rawat

**Affiliations:** 1 Department of Endangered Species Management, Wildlife Institute of India, Dehradun, Uttarakhand, India; 2 Faculty of Wildlife Sciences, Wildlife Institute of India, Dehradun, Uttarakhand, India; Feroze Gandhi Degree College, INDIA

## Abstract

It is of utmost importance to research on the spatial patterns of human-wildlife conflicts to understand the underlying mechanism of such interactions, i.e. major land use changes and prominent ecological drivers. In the north eastern part of India there has been a disparity between nature, economic development and fragmentation of wildlife habitats leading to intense conflicts between humans and Asian elephants (*Elephas maximus*) in recent times. Both the elephant and human population have increased in the past few decades with large tracts of forests converted to commercial tea plantations, army camps and human settlements. We analyzed data maintained by the wildlife department on human deaths and injuries caused by elephant attacks between 2006–2016 to understand spatial and temporal patterns of human-elephant conflict, frequency and distribution. The average annual number of human deaths and injuries to elephant attacks between 2006 to 2016 was estimated to be 212 (SE 103) with the highest number of such incidents recorded in 2010–2011. Based on a grid based design of 5 km^2^ and 25 km^2^ resolution, the main spatial predictors of human-elephant conflicts identified through Maxent presence only models are annual mean precipitation, altitude, distance from protected area, area under forests, tea plantations and agriculture. Major land use changes were assessed for this region from 2008 to 2018 using satellite imageries in Arc GIS and a predicted imagery of 2028 was prepared using Idrisi Selva. Based on the 2018 imagery it was found that forest area had increased by 446 km^2^ within 10 years (2008–2018) and the annual rate of change was 12%. Area under agriculture had reduced by 128 km^2^ with an annual (-) rate of change of 2.5%. Area under tea plantation declined by 307 km^2^ with an annual (-) rate of change of 12% whereas area under human settlements increased by 61 km^2^ with an annual (-) rate of change of 44%. Hotspots of human-elephant conflicts were identified in an east west direction primarily around protected areas, tea plantations and along major riverine corridors. During informal interactions with farmers, tea estate labors it was revealed that local community members chased and harassed elephants from agriculture fields, human settlements under the influence of alcohol and thus were primary victims of fatal interactions. Our analytical approach can be replicated for other species in sites with similar issues of human-wildlife conflicts. The hotspot maps of conflict risk will help in developing appropriate mitigation strategies such as setting up early warning systems, restoration of wildlife corridors especially along dry river beds, using deterrents and barriers for vulnerable. Awareness about alcohol related incidents and basic biology of elephants should be organized regularly involving non-governmental organizations targeting the marginalized farmers and tea estate workers.

## Introduction

Asian elephant (*Elephas maximus*) is listed as ‘Endangered’ by the IUCN, but the major threats to its survival still exists [1]. Habitat loss, fragmentation of elephant populations, human-elephant conflicts (HEC), and the illegal killing of elephants have adversely affected elephant conservation throughout its distribution range. HEC adversely affect local communities in the form of loss of human lives, damage to crops and property. Such incidents generate antagonism amongst local communities leading to retaliatory killings of elephant and undermining of conservation efforts. Thus understanding HEC is crucial in many sites where solutions to escalating conflicts are urgently required [2]. Knowledge of the spatial-temporal patterns of HEC help local government, wildlife officials, civil organizations plan mitigation measures accordingly.

Based on the latest population estimation exercise conducted by the Government of India, elephant population is estimated to be *ca*.27,000 [3] spread across an area of about 109,500 km^2^ in 23 states of India [1]. In some of these areas, an average of 400 humans have died annually due to elephant attacks over the last five years (2012–2017) (Project Elephant) while in retaliation, 40–50 elephants have been killed during crop-raiding by local communities [4]. Apart from loss of human and elephant lives, considerable amount of crop and property damage occur every year with the Federal and State Governments spending substantial funds in controlling such depredation events and paying ex gratia / compensation to affected people [5]. In India, protected areas encompass only 22% of the elephant habitat while the remaining are fragmented forests and agricultural areas. Around 30% of the elephant population exists within large contiguous forests. While the rest majority occur in smaller groups within highly fragmented landscapes with human densities at certain sites >500 persons per km^2^. With such extensive overlap with humans and shared resources these isolated elephant populations have lower chances of long-term survival and conflicts are inevitable in majority of these sites. One such fragmented landscape with a relatively small elephant population is North Bengal located in the north-eastern part of India sharing international borders with Nepal, Bhutan and Bangladesh. The recent elephant population in North Bengal is reported to be around 500 individuals spread across an area of 2000 km^2^ [3].

The North Bengal elephant population has been fairly well studied with research conducted on various aspects of their status, distribution, usage of corridors, activity and habitat utilization through satellite telemetry and field surveys [6–8]. Based on Lahiri and Choudhury [6], this region had presence of more bulls with a population size of 300 elephants recorded in the 1990’s [9]. North Bengal Landscape underwent severe change in the 18^th^ century when British planters cleared large tracts of forests to establish commercial tea plantations. Subsequently a large number of tribal people from Chotanagpur plateau, Central India were brought in by the British to work as daily laborers in these gardens [10]. After Independence and an aftermath of the Indo-China war in 1962, large number of army settlements were also established here clearing vast tracts of forest lands [10]. A review of existing literature suggests an initial reduction in elephant population followed by protection, creation of forest reserves and cessation of capture resulting in a healthy population [9]. Nonetheless, fragmentation of habitats continued with increasing human immigration from neighboring regions, ultimately occupying prime elephant habitats. This lead to a steady rise in HEC with majority of such incidents occurring outside protected areas [11]. According to published literature, primary drivers for regional land use/ land cover change are deforestation, rangeland modification, intensification of agriculture and urbanization [12]. Socio-economic and biophysical characteristics also contributed significantly towards land cover change [13,14].

Taking into account, such drastic land use change due to increase in anthropogenic activities and magnitude of HEC, we undertook this present study in North Bengal to understand the spatial patterns of HEC, identify hotspots to concentrate mitigation measures, and predict land use change. Our objectives were to document (a) spatial factors and landscape variables responsible for conflicts, (b) identify human activities which make them vulnerable to such incidents, and (c) identify hotspots of conflicts, and (d) assess major land use/land cover changes. For the present study, we did not investigate primary drivers of crop depredation and property damage by elephants. We used the results to provide guidelines to mitigate HEC and formulate future strategies.

## Material & methods

### Study area

**Fig 1:**
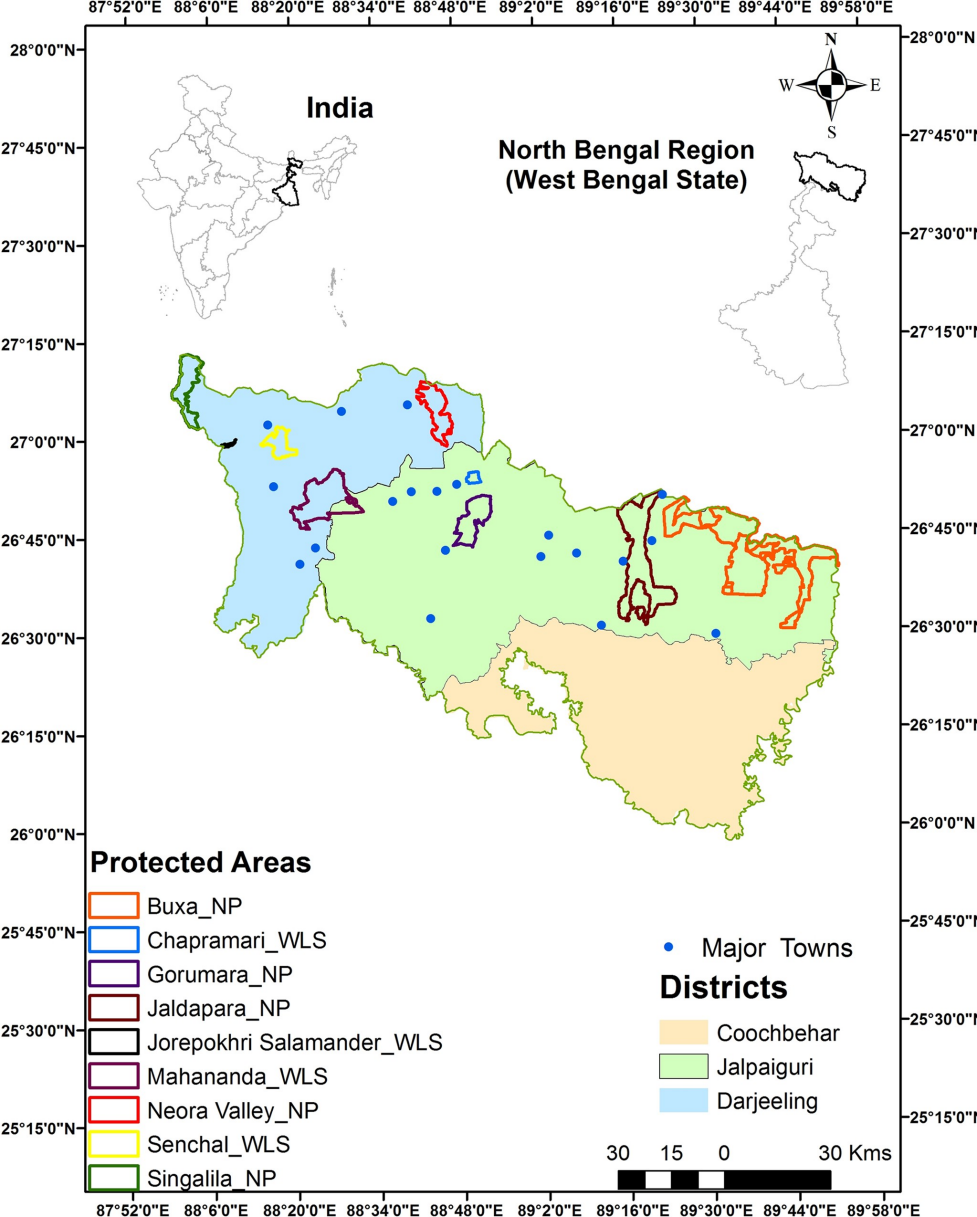
A Map of North Bengal showing the districts, wildlife protected areas, and important towns.

The present study was conducted across the entire North Bengal region comprising of three districts (administrative units) *viz*., Darjeeling, Jalpaiguri, and Coochbehar that are spread across an area of *ca*. 12,700 km^2^ (Fig 1). The region is dissected north-south by swift flowing rivers and alluvial floodplains that drain into the Brahmaputra-Gangetic delta. The major rivers of the northern Bengal region from the west are the Mechi, Teesta, Jaldhaka, Torsa and Sankosh. The Torsa river flowing between the Jaldapara Wildlife Sanctuary and Buxa Tiger Reserve is the boundary between Eastern Dooars and Western Dooars, while the Sankosh river is the border between West Bengal and Assam. The major rivers of this region are Teesta, Mechi, Torsa, Rydak and Sankosh. Most of these rivers are flood prone and some of the rivers change their course regularly, resulting in large area under alluvial floodplains. An average of (300–700) persons per km^2^ inhabit this region (Census 2011, https://www.census2011.co.in/census/district/1-darjiling.html, http://jalpaiguri.gov.in/html/census.html, https://www.census2011.co.in/census/district/3-koch-bihar.html accessed on July 2018) with the primary occupation being agriculture, livestock rearing and tea estate workers. This region receives an annual rainfall of 3160 mm.

This region is characterized by moist tropical and sub-tropical forests along foothills of the Eastern Himalaya and includes 3 National Parks and 2 Wildlife Sanctuaries. These include Buxa National Park and Tiger Reserve (761 km^2^), Jaldapara National Park (217 km^2^), Gorumara National Park (80 km^2^), Chapramari Wildlife Sanctuary (9.5 km^2^) and Mahananda Wildlife Sanctuary (158 km^2^). The other protected areas such as Neora Valley National Park, Singalila National Park and Senchal, Jorepokhri Wildlife Sanctuaries are located beyond 1000 meters altitude in the higher reaches of the Eastern Himalayan region. This entire stretch of forests along the foothills of North Bengal from the Indo-Nepal border with Mechi river in the west to the Sankosh river in the east bordering Assam is believed to be a historically contiguous elephant range. The major mammalian fauna of this region are the endangered one-horned rhinoceros (*Rhinoceros unicornis*), gaur (*Bos gaurus*), sambar (*Rusa unicolor*), chital (*Axis axis*), rhesus macaque (*Macaca mullata*) and a host of diverse fauna and flora with leopard being the apex predator and only large carnivore present [15]. Apart from elephants, this region also experiences one of the highest human-leopard conflicts in India [16].

### Field methods

#### Data collection and field visits

The data were collected in collaboration with the West Bengal state Forest Department, the authority that granted permission for the study. The state forest staff accompanied the research team and assisted in collecting details of the conflict incidents. We obtained verbal approval from the local community heads/ village headmen and labor heads of the tea gardens for conducting the study. We inquired about the details of such incidents from family members of conflict victims, companions, local people and forest personnel who were present or had visited these sites after the incidents happened. A person was interviewed only after verbal consent was taken and once they agreed to share the details, they also accompanied us to the sites.

The department had a register where such events were recorded for payment of ex gratia / compensation to the victims. We compiled data from the West Bengal state forest department compensation records between 2006–2016, reviewed literature available, and newspaper reports regarding incidents of elephant attacks on humans. Based on this secondary data, we documented nature of such incidents and extant of HEC. We also made field visits (N = 234) to reported HEC sites where elephant attacks had led to human injuries and/or death in the last 10 years between November 2016- June 2018. Our field visits were conducted across the 3 administrative districts during which we took assistance of local forest personnel, police, community heads, tea estate managers and local field assistants. All these conflict locations were later mapped in Arc GIS 10.2.2. During such field visits, we interviewed family members, neighbors, companions of victims who were present during the attacks and inquired about details such as age and occupation of victim, time and month of attack, activity during attack, vegetation type and altitude of conflict site as well as whether the attack resulted in death or injury (S1 Appendix). For this purpose, we obtained verbal approval from the local community heads/ village headmen and labor heads of the tea gardens for conducting the study. Due to the large number of crop raiding incidents and lack of a standardized protocol to estimate crop damage we did not record such events.

#### Vegetation mapping

To prepare major land use/land cover map, we downloaded Landsat 5TM and Landsat 7 satellite imagery of North Bengal for the year 2008 (April imagery) with 30 m resolution. To document changes in land use patterns we also downloaded 2018 imagery using Landsat 8TM March imagery for the same region. The path ids, sensor and number of bands in the imagery are provided in (Table 1). To improve clarity of the downloaded satellite imageries, we used a combination of layer stacking, mosaic and subset through ERDAS Imagine 14. We tried radiometric correction, histogram match and utilization but due to incompatibility and mix match of different tiles for the study site we used unsupervised classification over supervised technique. We also did ground verification and collected (N = 700) locations on major land use types of the study site between October 2017- May 2018. We used a total of 80 classes initially for unsupervised classification and started allotting respective values to each pixel sample. Once each pixel was allotted we used thematic recoding to combine all pixels of a single field to one.

**Table 1:** Satellite Imagery used with sensor, path row and number of bands used for major land use classification.

Satellite	Sensor	Path row	Bands
LANDSAT 5	MSS & TM	138 41, 138 42, 139 41, 139 42	**7**
LANDSAT 8	OLI & RIRS	138 42, 139 41, 139 42	**11**

#### Conflict risk mapping

We analyzed the data collected during field visits to derive about socio-temporal pattern of attacks. Chi-square test (α = 0.05) [17] was used to compare attacks between seasons, time zones, months, different age classes and major occupation of people. The study area was initially stratified into 5 km^2^ grids using Arc GIS 10.2.2. Apart from the fine scale resolution, we were also interested to identify spatial factors at coarser scales, thus the analysis was conducted for (5*5) i.e. 25 km^2^ grids. There were a total of 600 grids available for 25 km^2^ and for 5 km^2^ grid it was 2780.

We selected a total of 14 predictor variables based on their ecological importance to model HEC risk (Table 2). Area of major land use types were derived from the classified imagery of 2018. We extracted length of road and major rivers for each individual grid using the Roads and Drainage layers obtained from the Digital Chart of the World [18]. For each grid centroid, we generated altitude using the 90 m spatial resolution digital elevation maps [19]. Annual mean temperature and precipitation were derived from World BIOCLIM data [20]. Using online human census data 2011 of India available at the resolution of a village, human footprint data was derived for every grid whereas night light data was extracted using 1000 m spatial resolution night-time visible lights data of India [21]. To tabulate distance from protected areas, we used the tool Euclidean distance for every grid. Once the grid files were finalized, we clipped all the variables to 5 and 25 km^2^ grids. All the fourteen predictor variables were converted to raster files (ASCII format) in Arc GIS 10.2.2 for both 5 and 25 km^2^ grids. After compiling these open source variables, we used a total of (N = 228) conflict locations as sample data to run presence only models and predict hotspots of elephant-human conflicts using Maxent program. Maxent is a common species distribution modelling program used to predict distribution of species/events based on presence records and set of environmental predictor variables [22].

**Table 2:** Major predictor variables considered for conflict risk mapping.

Serial No	Predictor Variables
1.	Annual Mean Temperature
2.	Annual Mean Precipitation
3.	Length of rivers
4.	Length of roads
5.	Distance from Protected Areas
6.	Area under Forest
7.	Area under Agriculture
8.	Area under Tea Plantations
9.	Area under Human Settlements
10.	Area under river/water bodies
11.	Area under Sand Bed
12.	Altitude
13.	Human Footprint
14.	Nightlight Data

Maxent modeled probability of conflict within each 5 and 25 km^2^ grids as a function of all the independent predictor variables. A higher value computed for a grid indicated higher probability of conflict and vice versa. The final computed model was a probability distribution over all the grid cells. Response curves were created for each predictor variable and we used jackknife to measure variable importance in the final modeled output. We used a total of 25 locations as random test data or training data to evaluate the final model performance. We specified a total of 5 replicates which allowed the model to run multiple times ultimately averaging the results from all models created. To ensure adequate time for convergence and robustness in the final model output, we used a total of 500 iterations. As prevalent with other presence-pseudo absence methods, Maxent generates a background or pseudo-absence sample of points which by default are 10000 randomly selected from the entire study site [23].

Table 2. Major predictor variables considered for conflict risk mapping.

#### Land use/land cover change detection

To detect changes in major land use classes of North Bengal we ran markov and cellular automata markov model (CA Markov) in Idrisi Selva [24] using the unsupervised imageries of 2008 and 2018. Markov model is a process in which the state of the system at time 2 can be predicted based on the current state of the system at time 1 given a matrix of transition probabilities from each cover class to every other cover class is available. Markov models produce multiple outputs such as a i) transition probability matrix which expresses the likelihood that a pixel of a given class will change to any other class or stay the same in the next time period ii) A transition areas matrix which expresses the total area in cells expected to change in the next time period and iii) a set of conditional probability images, one for each land cover class. These maps express the probability that each pixel will belong to the designated class in the next time period. They are called conditional probability maps since this probability is conditional on their current state. The most crucial spatial element underlying dynamics of change events is proximity i.e. areas which are near to existing areas of a particular class will in majority exhibit the same class. These patterns can be conveniently modeled using cellular automata. Cellular automation is a cellular entity which varies based on its previous state and immediate neighbors with the major difference being application of a transition rule depending not only upon the previous state but also on the neighborhood attributes. Thus based on this mechanism we generated a predicted major land use imagery of North Bengal for the year 2028.

## Results

### Human deaths and injuries, crop and household damage

Out of a total of (n = 2122) incidents, 476 persons died whereas the rest (n = 1646) humans sustained injuries due to elephant attacks between 2006–2016. The annual mean number of humans killed and injured by elephant attacks in North Bengal was estimated to be 47 (SE 8) and 164 (SE 97) respectively (S1 Fig). The highest number of attacks was recorded in 2010–11 (360 injuries and 56 deaths). There was a sudden peak in number of such incidents between 2008 and 2012 which gradually declined. Between 2006–2016, annual crop damage by depredation events were estimated to be 2078 hectares (SE 1096). Based on complaints registered, the state forest department officials paid an annual sum of INR 4701309 (= US$ 67479), (SE INR 1759179) for compensating human deaths and injuries whereas for crop damage it was estimated to be or INR 5417390 (= US$ 78930), (SE INR 2768212) per year. The annual compensation due to property damage based on forest department records were estimated to be or INR 3008012 (= US$ 43826), (SE INR 1320804). Thus the annual *ex gratia* / compensation amount paid by the forest department regarding human death, injury, crop depredation and household damage combined was US$ 190864 or INR 131 lacs (SE 47 lacs). Data on elephant deaths due to public retaliation during crop raiding and household damage, human injury and death were not maintained by the forest officials and thus are not reported.

#### Seasonal and temporal variation of elephant attacks

Based on the field visits to (N = 228) conflict sites, 54% of elephant attacks occurred between May-July (30%) and August-October (24%) followed by 32% between November to January and rest 14% during February to April (*χ^2^ =* 8.54, df = 3, p < 0.05) (S1 Fig). Fifty percent of these attacks were recorded between 1800h and 0000h (23% between 1800h and 2100h and 27% between 2100h and 0000h) whereas 14% between 0000h and 0300h and 13% between 0030h and 0600 h respectively (*χ^2^* = 39.73, df = 7, p-value < 0.05). Majority of the elephant attacks occurred in flat areas with an average elevation of 103 m (SE 37).

#### Age, profession and activity of victims

Majority of the elephant attack victims were middle aged adults, with 20% in the 20–30 years category followed by 20% in the 30–40 years, 23% in the 40–50 years and 16% in the 50–60 years category respectively. Seventy-four percent of the elephant attacks victims were males. Thirty percent of the victims were farmers, 19% daily labors and 17% tea estate workers by profession. During information interactions and inquiry, family members, companions and colleagues of victims responded that 36% of them were drunk and were chasing elephants in agriculture fields and near households, 20% were returning home after dark from work, 7% had gone collecting fuelwood from the forests, 8% were defecating in the open at night and 8% were sleeping inside houses when attacks occurred (*χ^2^ =* 38.57, df = 5, p < 0.05). Most of the victims were in a group comprising of < 3 people with an average of 4 households present in the vicinity of the site, and 25% of the attacks occurred in patches dominated by miscellaneous tree species, 21% near betel nut plantations, 13% within tea estates and 12% in agricultural fields (χ^2^ = 43.59, df = 8, p < 0.05).

#### Major land use change detection and prediction

Based on ground knowledge, Google Earth and combining identical pixels we identified 6 major land use classes for both 2008 and 2018 Figs 2 & 3 (Table 2). Accuracy of the major land use class map was estimate to be 82% and 86% respectively for 2008 and 2018. Major changes were detected between 2008 and 2018 for certain classes such as forests, human settlements and tea plantations. Human settlements increased by 44% in the last 10 years’ probably due to influx of people from neighboring Bangladesh and Nepal and rapid urbanization. The predicted area estimates under major classes for 2028 imagery (S2 Fig) indicates a marginal rise in tea plantations, riverine patches and sand beds compared to the present scenario in 2018 (Table 3).

**Fig 2:**
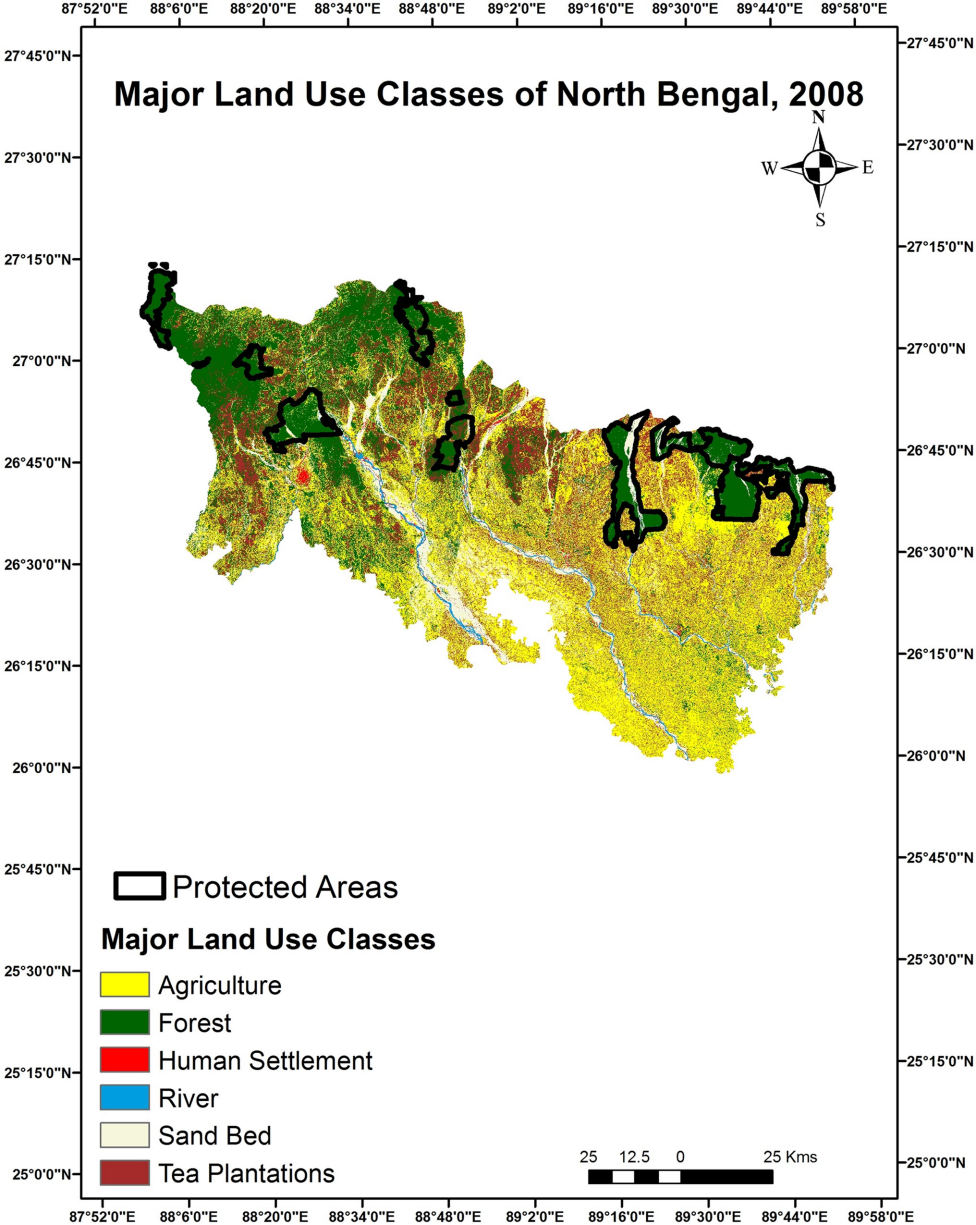
Major land use classes of North Bengal region 2008.

**Fig 3:**
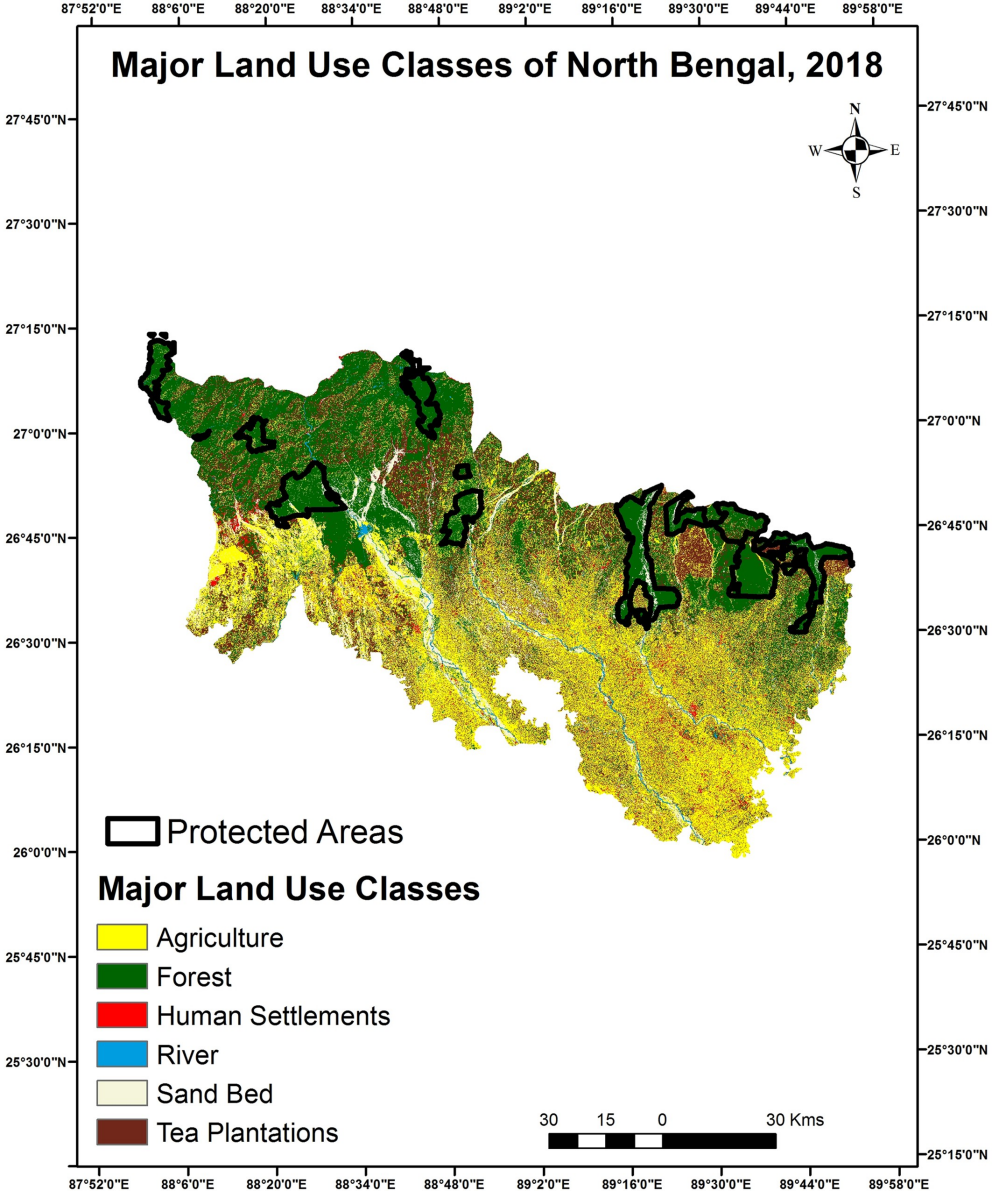
Major land use classes of North Bengal region 2018.

**Table 3:** Major land use classes by area for North Bengal (2008–2018).

Major Land Use Class	Area (km^2^) (2008)	Area (km^2^) (2018)	Change in last 10 years %	Predicted Area (km^2^) (2028)
Forest	3733.35	4178.97	11.9	4023.01
Agriculture	5118.03	4990.21	-2.5	4889.19
Tea Plantations	2458.31	2151.26	-12.49	2377.7
Human Settlements	137.98	199.14	44.32	166.97
River	115.65	125.14	8.20	134.18
Sand Bed	1111.39	1030	-7.32	1083.64

#### Predictor variables

A total of 141 and 47 locations were used for training and testing for 5 km^2^ grids whereas 138 locations and 46 locations were used for training and testing for 25 km^2^ grids. After model convergence and averaging for 5 replicates, predictor variables such as i) Mean Annual Rainfall ii) distance from protected areas iii) altitude iv) area under agriculture and v) sand/dry river beds were ecologically significant variables affecting human-elephant conflicts in North Bengal on a scale of 5 km^2^. Receiver operating characteristic curves (AUC) values for both 5 and 25 km^2^ models were 0.923 and 0.926 respectively (S3 Fig). For 5 km^2^ grids, the predictor variable with highest gain when used in isolation is mean annual rainfall, which therefore appears to have the most useful information by itself. The predictor variable that decreased the gain the most when omitted was distance from protected area, which therefore appears to have the most information that isn't present in the other variables. Based on the response curves generated through Maxent, probability of conflicts decreased with increasing altitude, increase in area under agriculture and distance from protected areas whereas it increased with annual rainfall to a certain extent and area under forests. Probability of conflicts peaked in the initial and final stages with increase in area under dry river bed/sand and remained constant for the rest.

For a coarser scale of 25 km^2^, variables such as i) Mean Annual Rainfall ii) area under forests iii) altitude iv) area under tea plantations and v) distance from protected areas were major ecological drivers of conflicts. The predictor variable with highest gain when used in isolation was Mean Annual Rainfall, which appeared to have the most useful information by itself. Response curves of major predictor variables depicted that probability of human-elephant conflicts increased with mean annual rainfall, area under forests and area under tea plantations whereas it declined with altitude and distance from protected areas. Probability of conflicts increased with a rise in area under agriculture till a certain extent and then dipped.

#### Spatial pattern of human-elephant conflicts

**Fig 4:**
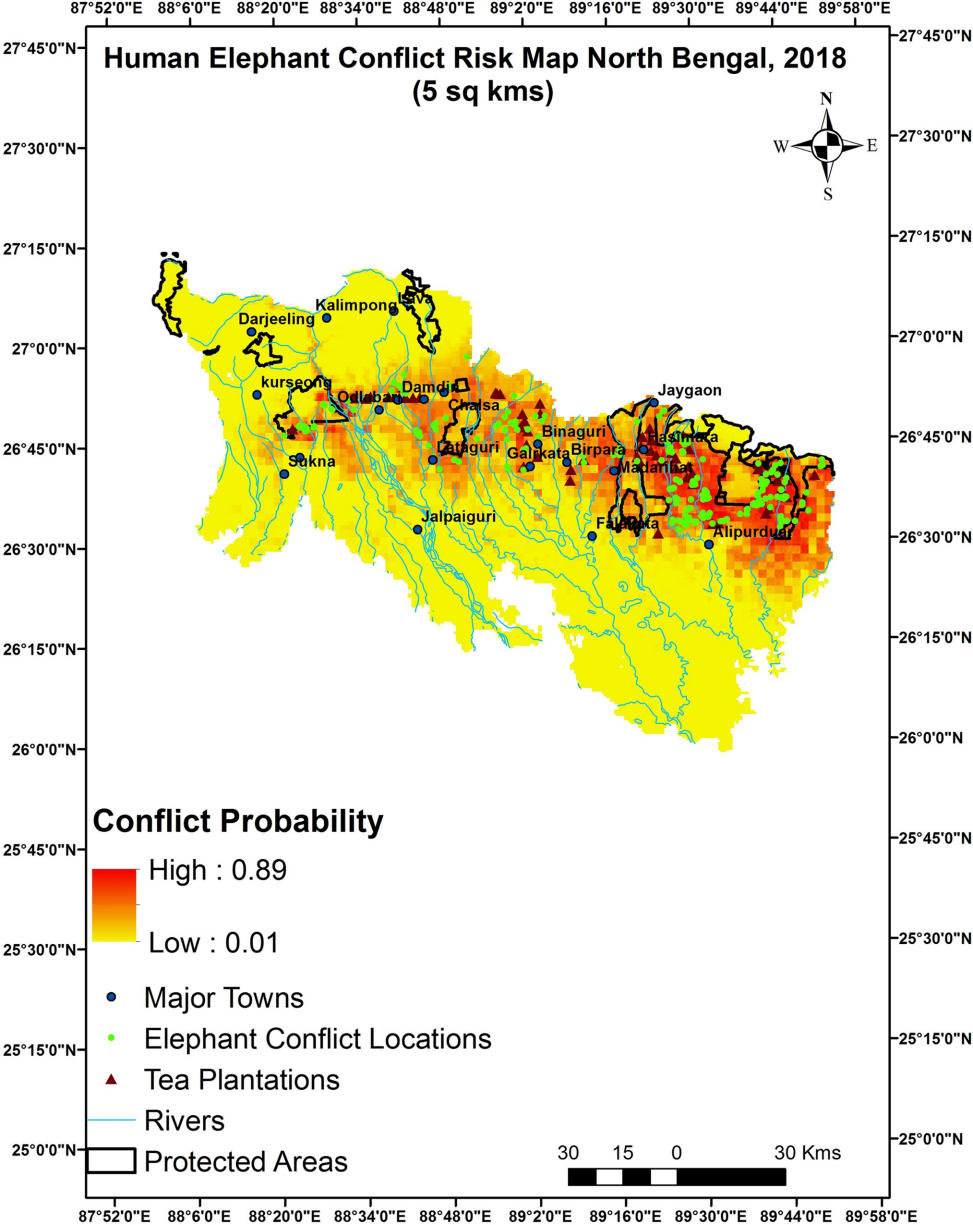
Predictive map of human-elephant conflict hotspot on 5 km^2^ resolution, North Bengal India.

**Fig 5:**
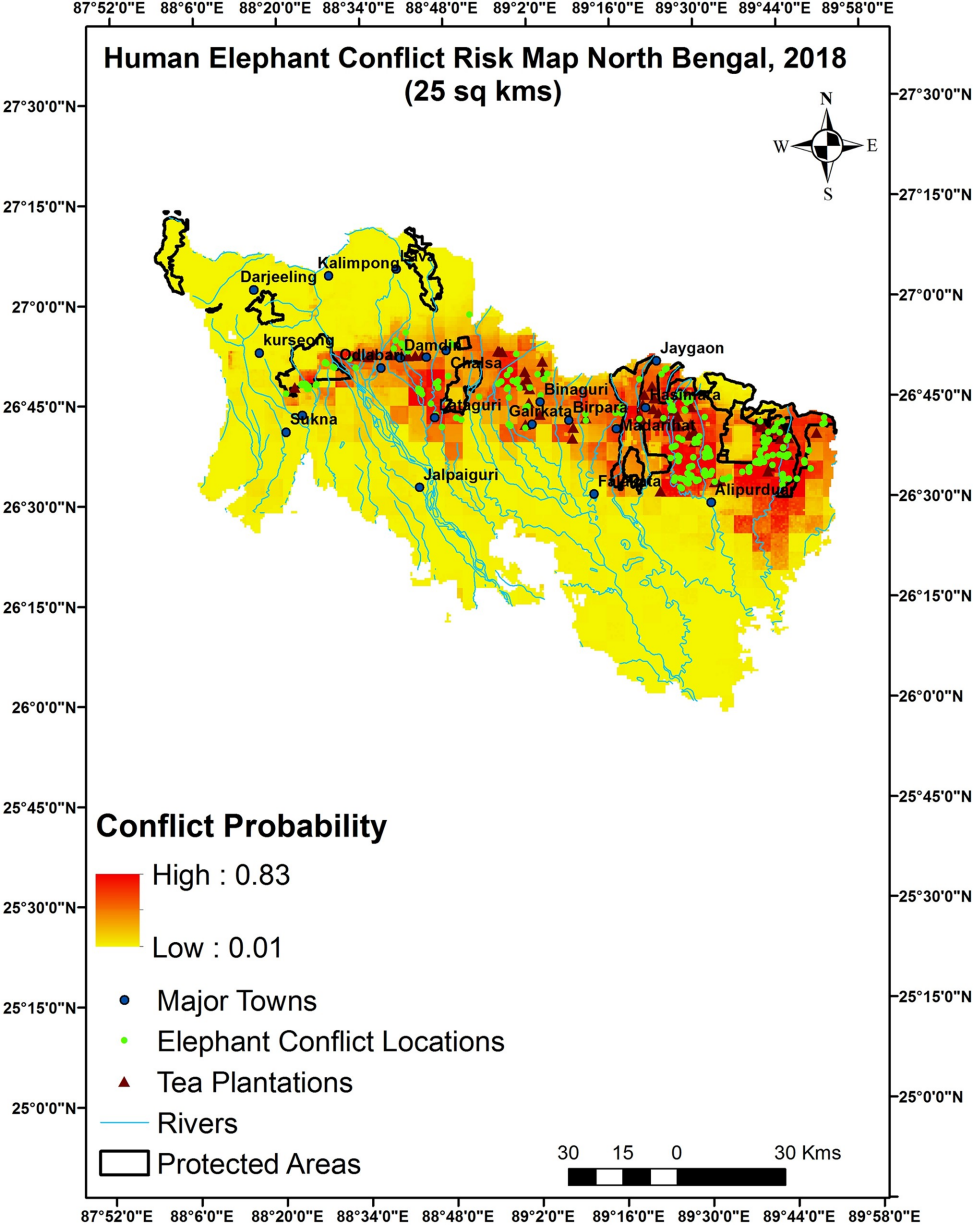
Predictive map of human-elephant conflict hotspot on 25 km^2^ resolution, North Bengal India.

Predictive maps for both 5 and 25 km^2^ grids depicted eastern and central regions of North Bengal as HEC risk hotspots Figs 4 & 5. The sites around peripheral areas around Buxa Tiger Reserve and Jaldapara National Park in the east and Gorumara National park, Chapramari WLS in the central part and major tea plantations in North Bengal showed higher probabilities of conflicts compared to the northern, southern and western part of the landscape. This part also has a number of townships, army settlements and a 160 km long broad gauge railway track.

## Discussion

The present study is a pioneering initiative to compare and explore the spatial patterns of HEC for the North Bengal landscape. Different ecological and spatial drivers were identified at finer and coarser scales using a systematic grid-based approach. The study also provided a major overview of dominant land use types, its effect on the prevailing intensity of HEC and a predicted change in the next 10 years. Based on government records, the total number of human deaths due to elephant attacks in India ranged between 390–420 between 2013–2016 [25] whereas the current All India population estimate for elephants are reported to be 27,312 [3]. According to published results of this nationwide exercise, North Bengal elephant population has been estimated to be 488, i.e. only 1.8% of the entire elephant population of India. But according to the number of human deaths registered with the government, 7–12% of the elephant related conflicts in the entire country occurred in North Bengal region between 2013–2016. Thus to summarize this, 1.8% of the elephant population of India was responsible for 12% of the human deaths confirming to the magnitude of the problem. Apart from the elephant population, this region also supports a high human density of 500–700 persons per km^2^ (Census 2011). There are also reports of elephants crossing over to North Bengal from neighboring Assam state regularly confirming seasonal usage of these areas [8]. Due to submergence of forest areas during floods in the Brahmaputra valley between June-September, elephants might be moving into the North Bengal landscape during monsoons and this could also trigger conflicts with humans. Findings of previous studies conducted, indicate a higher proportion of bulls and a sex ratio of 1:1.6 for male and females for the elephant population here [8]. Elephants being a long lived species with a long gestation period, small number of young ones born and low calf survival [26], the sex ratio and proportion of bulls in the population will not have changed drastically over a period of 10–15 years. Adult bulls are reported to raid crop fields and cause more frequent conflicts with humans compared to female herds in most regions of Asia and Africa [26–28] and could be one major reason for such magnitude of HEC in this region.

Elephant clans comprising of females and sub adults, young ones are more likely to abandon present home ranges when experiencing stress due to draught, severe poaching, overpopulation, severe human disturbance and degraded habitat and disperse in search of new territories breaking down into smaller groups. Extant of home ranges are an indication of resource availability with the largest i.e. 600 km^2^ recorded for Asian elephants from Southern India for female herds while for males it has been reported to be 400 km^2^ from northern India. A study in 2003 documented average home ranges to be 580 km^2^ for female elephant herds and 300 km^2^ for males in North Bengal indicating low availability of resources [8].

North Bengal underwent major changes in the 19^th^ century when large tracts of forests were felled to establish tea plantations. Subsequently post partition in 1947 and in 1971 after formation of Bangladesh there was an influx of immigrants and the state government cleared additional forested areas to accommodate them [29]. The neighboring state of Assam also lost a total of 1800 km^2^ of lowland evergreen forests since 1970 primarily to settle such large number of immigrants and resident elephant populations were displaced from such areas [30]. Members of these displaced elephant populations are reportedly stressed and are as aggressive as bulls [31] and could be a primary reason of aberrant behavior of elephants. Major land use change detection undertaken within this study reported an increase of 44% area under human settlements between 2008 and 2018 with a slight increment of 12% forest area due to compensatory afforestation and plantation programs undertaken. Area under dry river/sand bed declined by 8 percent which could be due to encroachment by human settlements and other developmental works. With the establishment of Teesta barrage in 1980’s near Siliguri town, the traditional migratory route of elephants from east to west was disrupted and prime riverine patches were lost due to submergence by the surplus dam water. Elephants spend substantial time in the prime riverine patches and sand beds and dams often disrupt their traditional routes, causing habitat loss and fragmentation [26]. Nepal Government constructed an 18 km long electric fence along the border with West Bengal, India in to stop elephants from entering Nepal and this has further disrupted their migratory route westwards and confined them to a smaller region [32]. All these factors must have led to severe fragmentation of wildlife habitats, degradation of forest patches, probable disintegration of larger clans into smaller groups of elephants ultimately escalating frequency of conflicts in this region.

The major spatial predictors of human-elephant conflicts on a finer scale were distance from protected areas, altitude, mean annual rainfall, area under agriculture and sand bed whereas on a coarser scale area under tea plantations was also included. Mean annual rainfall is suggestive of areas with higher crop biomass which are inherently conflict prone due to frequent visitations by elephant herds. Studies conducted in Africa have found that elephants continue to cause damage in the same areas every year confirming to a spatial patterns and a repetitive behavior in spite of large scale changes in land use patterns [33] and thus the hotspots predicted in the present study will continue being high risk zones in the future. Clustering of crop damage locations are indicative of the long memories of elephants and usage of traditional routes [34] and such patterns might be there even with human injuries and deaths. Our results also depict spatial clustering of such events. Probability of conflicts decreased with increasing altitude which was expected of a species preferring flat lands, grasslands and low land forests. Though there are occasional reports of elephants involved in crop raiding even at 1000 meters but those are rare events with hardly any human fatalities reported till date. There are more than 450 tea plantations within this region with 60% of these estates located in the eastern, central and western part [35]. As depicted by the hotspot maps, conflicts are also confined primarily to this tea growing zone which suggests that such areas are barriers to elephant traditional migration routes. A considerable extent of historic elephant refuges has been lost to tea plantations which have further fragmented the present habitat.

The frequency of conflicts increases during the rainy season which also coincides with the harvest of major agricultural crops such as wheat, maize and paddy similar to findings of [8]. Now North Bengal being a resource limited and fragmented habitat, it is compulsive of elephants to seek nutritious agricultural crops as supplement [8]. Previous studies in South East Asia and Africa have also reported that conflicts intensify during monsoons when elephants raid agriculture fields for nutritious and palatable crops thus confronting humans more frequently. Despite availability of resources within protected area and forests during monsoon, elephants seek out accessible and abundant food in the form of crops [36]. Majority of the incidents happened after dark when people were returning from work or driving elephants from agriculture fields and near human habitations. Inhabitants of this region grow a lot of horticultural crops such as jackfruit, mango and betel nut trees around households and a major proportion of the incidents also occurred when elephants came to feed on these crops at night. During informal interactions and interviewing people in North Bengal, respondents reported that elephants regularly damage households to seek out stored grains, paddy and locally brewed alcohol called “*haaria*”. The intensity of crop raiding by elephants also could be one of the major reasons for conflicts happening around households and agriculture fields similar to findings of a previous study [8]. Even in Karnataka southern India majority of human deaths by elephants occurred within coffee, cardamom, areca nut and coconut plantations [37]. More than one third of the victims to have died of elephant attacks were drunk and were chasing elephants which was similar to the findings of a study conducted in Malaysia [38]. Based on direct opportunistic encounters, lot of sightings of elephants especially of bulls and smaller herds happened around dusk near forest and village interface while conducting field work. Telemetry data also suggests that elephants here are more nocturnal [8] and could be one of the principal factors of conflicts occurring at night. Even in neighboring Orissa state elephants have been observed to take refuge in the forest patches during the day and raid crops at night [1].

Previous studies concluded that elephants do not discontinue using high-risk human settlements which were once part of their original home range and instead modify their activities by travelling faster or being nocturnal within such areas [39,40]. Human mortality and injury around human settlements and agriculture areas are primarily due to i) continued harassment and taunting of elephants while being driven back to forests from crop fields or ii) the frustration of being obstructed by local communities, farmers while they try reaching the fields during and lastly iii) by getting too close or getting involved in provoking activities to already injured, traumatized, harassed animals or males in musth or females with young calves [26, 41].

There is also presence of large number of human settlements primarily of daily wage labors working in these gardens and they regularly chase and harass elephants. Also there are wildlife squads comprising of forest officials and village community members, specially trained kunki elephants who drive wild elephants from one region to the other thus aggravating the problem. Peripheral regions around protected areas were prone to conflicts with the probability declining with increasing distance from PAs’ similar with findings of other studies [8, 42–44, 38]. Considering that a herd of elephants need a contiguous habitat patch of 250–300 km^2^ for sustenance [45], the average size of protected areas in this region are l10 km^2^ which is not substantial enough to support a sizeable population.

To resolve this problem, habitat restoration measures such as improving condition of wildlife corridors should be initiated to maintain gene flow and allow animals to freely move across the landscape. Historically, North Bengal was a contiguous landscape with elephants moving across neighboring Nepal in the west, Bhutan in the north east, Assam to the east and Bangladesh down south and this should be restored to ensure functioning of a meta population across this region [46]. Transboundary conservation initiatives between India, Bhutan, Nepal and Bangladesh should be taken up keeping in priority movement of elephants and stepping stones between protected areas such as riverine patches and dry sand beds should be conserved. Major anthropogenic activities should be restricted within such sites and artificial barriers to elephant movement such as construction of walls, dams and railway tracks, highways should be restricted. Majority of the tea estate labor colonies are located in an east west direction which is the major traditional route used by elephants and hence they can be shifted or reorganized in a north-south orientation. Considering the overabundance in elephant population within this relatively small landscape, reproductive control of females should be initiated. Short term measures such as using early warning systems, deterrents, growing unpalatable crops such as ginger and chilies, patrolling at night should also be explored. Government programs such as formation of village response teams along vulnerable zones to reduce conflicts should be advocated instead of just paying compensation to damage and casualty. Awareness camps regarding alcohol abuse and prohibition on availability of alcohol within the conflict hotspots can be taken up in collaboration with non-governmental organizations and district administration authorities. Elephants are long ranging species and confining them to fragmented forests will only aggravate the problem, thus tea estate owners and management authorities should be discouraged from harassing and allowing smooth passage of elephants. The vulnerability map of North Bengal will help in developing, deploying additional mitigation measures in the predicted hotspots and similar approach can be replicated in sites with issues of human-wildlife conflicts.

## Supporting information

S1 Fig Seasonal and temporal patterns of human-elephant conflicts, annual human injuries and deaths 2006–2016. (TIFF)

S2 Fig Predicted land use imagery for 2028, North Bengal. (TIFF)

S3 Fig Receiver Operating Curve (ROC) for 5 and 25 km^2^ Maxent models. (TIFF)

S1 Appendix Questionnaire used during the field visits to human-elephant conflict sites in North Bengal. (DOCX)

S1 Dataset Secondary data of wildlife wing, West Bengal Forest Department 2006–2016. (XLSX)
