# Supplementary figures and images for: Assessment and prediction of spatial patterns of human-elephant conflicts in changing land cover scenarios of a human-dominated landscape in North Bengal
Source: PLoS One. 2019 Feb 1;14(2):e0210580. doi: 10.1371/journal.pone.0210580 (PMC6358066; doi:10.1371/journal.pone.0210580)

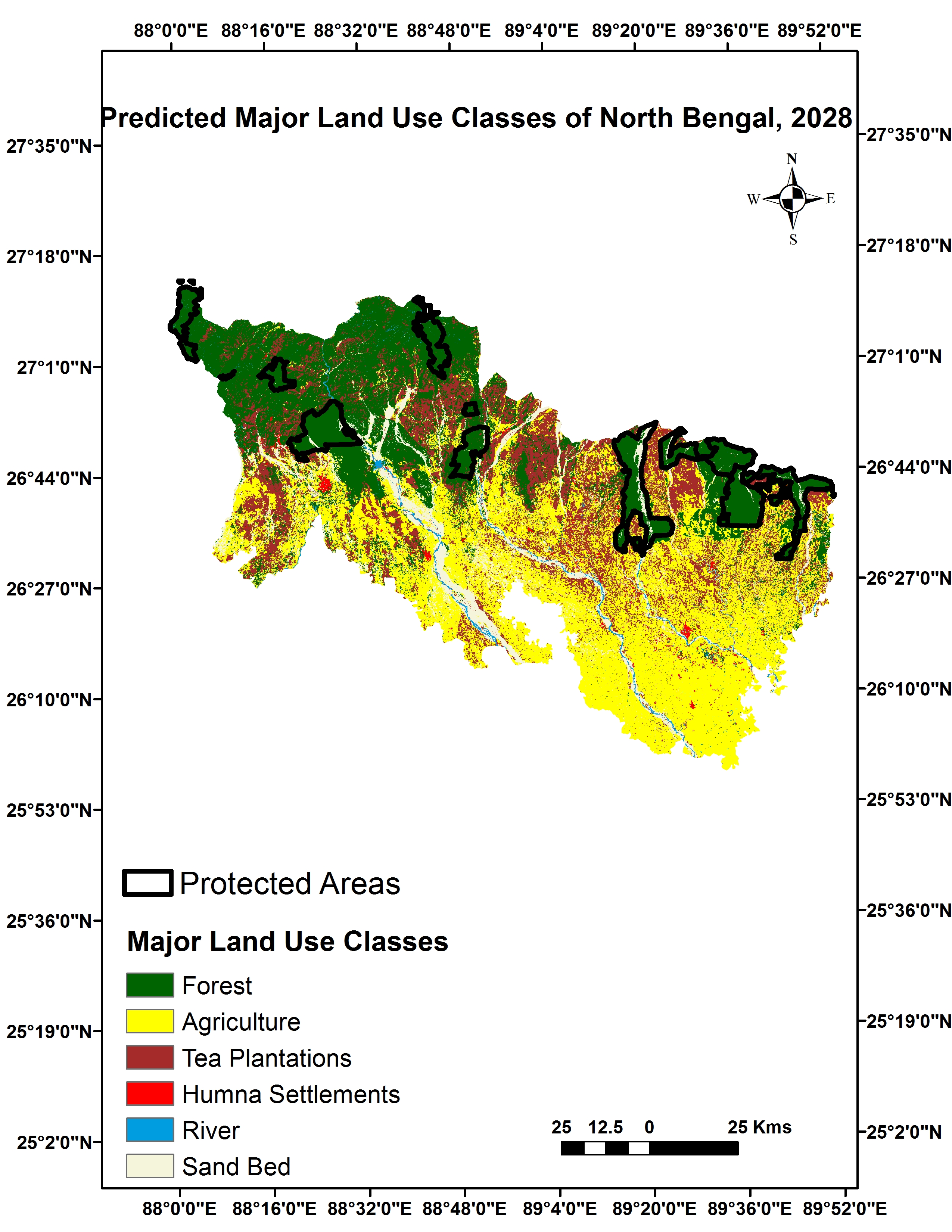

Supplement: S2 Fig — (TIFF) [file pone.0210580.s002.tiff]

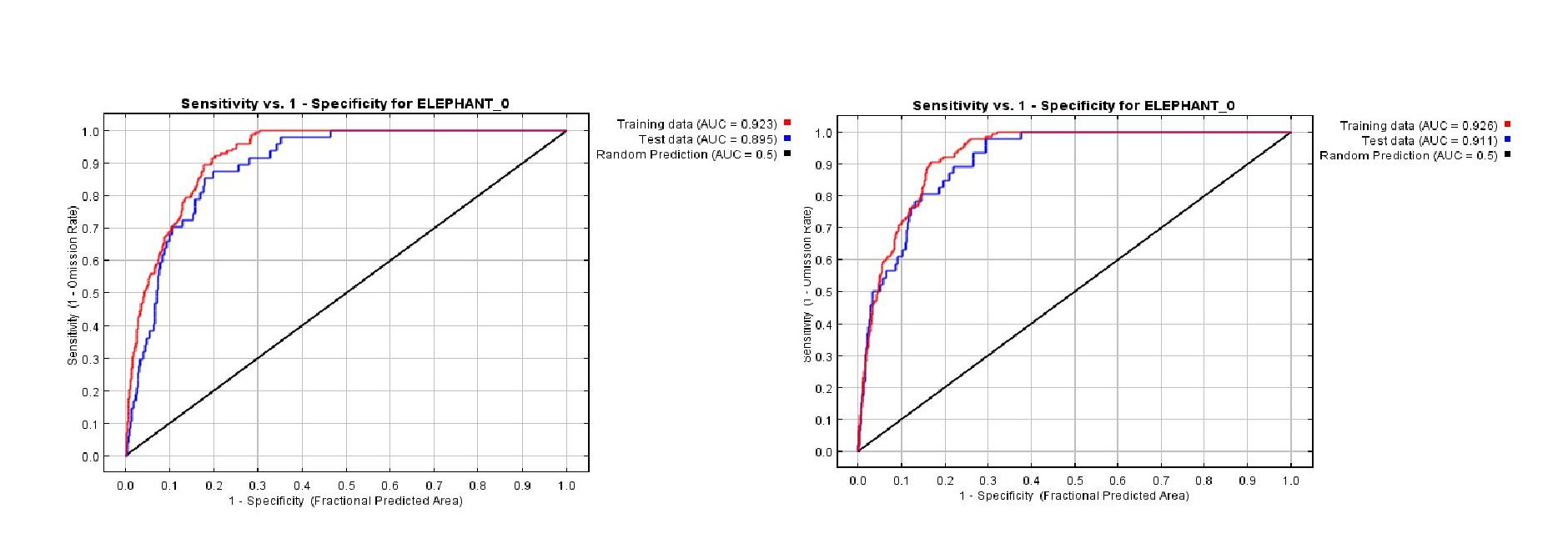

Supplement: S3 Fig — (TIFF) [file pone.0210580.s003.tiff]
