# Supplementary material for: Assessment and prediction of spatial patterns of human-elephant conflicts in changing land cover scenarios of a human-dominated landscape in North Bengal
Source: PLoS One. 2019 Feb 1;14(2):e0210580. doi: 10.1371/journal.pone.0210580 (PMC6358066; doi:10.1371/journal.pone.0210580)
Supplement: S1 Appendix — (DOCX) [file pone.0210580.s004.docx]

**Datasheet for human-elephant conflict details**

**Observer’s Name: _____________________ Date: ______________**

**Locality: _________________________ Village Name: __________________________________ District: _________________**

**Nearby PA: __________________________________ Forest: ____________ Division/Range/Beat: ______________**

| **GPS Loc.** | **Species** | **Altitude** | **Terrain type** | **Name and condition of victim** | **Age**  **& Gender** | **Occupation** | **Death/Injury** | **House damaged/No of persons nearby** | **Date &Time of Incident** | **Dominant vegetation**  **Type** | **Number of households in vicinity** | **Whether victim**  **had possession**  **of any object or**  **whether drunk/**  **Major activity** | **Whether accompanied by people or alone** | **Major Activity during attack** |
| --- | --- | --- | --- | --- | --- | --- | --- | --- | --- | --- | --- | --- | --- | --- |
|  |  |  |  |  |  |  |  |  |  |  |  |  |  |  |
|  |  |  |  |  |  |  |  |  |  |  |  |  |  |  |
|  |  |  |  |  |  |  |  |  |  |  |  |  |  |  |
|  |  |  |  |  |  |  |  |  |  |  |  |  |  |  |

Remarks: Mention about presence of guard and domestic dog:
